# Effects of Dextran-Coated Superparamagnetic Iron Oxide Nanoparticles on Mouse Embryo Development, Antioxidant Enzymes and Apoptosis Genes Expression, and Ultrastructure of Sperm, Oocytes and Granulosa Cells 

**DOI:** 10.22074/ijfs.2020.6167

**Published:** 2020-10-12

**Authors:** Azizollah Bakhtari, Saeedeh Nazari, Sanaz Alaee, Elias Kargar-Abarghouei, Fakhroddin Mesbah, Esmaeil Mirzaei, Mohammad Jafar Molaei

**Affiliations:** 1 Department of Reproductive Biology, School of Advanced Medical Sciences and Technologies, Shiraz University of Medical Sciences, Shiraz, Iran; 2 Department of Anatomy, Faculty of Medicine, Hormozgan University of Medical Sciences, Bandar Abbas, Iran; 3 Department of Anatomical Sciences, School of Medicine, Shiraz University of Medical Sciences, Shiraz, Iran; 4 Department of Medical Nanotechnology, School of Advanced Medical Sciences and Technologies, Shiraz University of Medical Sciences, Shiraz, Iran; 5 Faculty of Chemical and Materials Engineering, Shahrood University of Technology, Shahrood, Iran

**Keywords:** Apoptosis, Nanoparticles, Oocytes, Oxidative Stress, Spermatozoa

## Abstract

**Background:**

Although application of superparamagnetic iron oxide nanoparticles (SPIONs) in industry and medi- cine has increased, their potential toxicity in reproductive cells remains a controversial issue. This study was under- taken to address the response of sperm, oocyte, and resultant blastocyst to dextran-coated SPIONs (D-SPIONs) treat- ment during murine *in vitro* fertilization (IVF).

**Materials and Methods:**

In this experimental study, murine mature oocytes were randomly divided into three groups: control, and low- and high-dose groups in which fertilization medium was mixed with 0, 50 and 250 µg/ml of D- SPIONs, respectively. Sperm and/or cumulus oocyte complexes (COCs) were cultured for 4 h in this medium for elec- tron microscopic analysis of sperm and COCs, and assessment of developmental competence and genes expression of *Gpx1, Sod1, catalase, Bcl2l1* and *Bax* in the resultant blastocysts.

**Results:**

Ultrastructural study of sperm, oocyte, and granulosa showed destructed mitochondria and membranes in spermatozoa, vacuolated mitochondria and distorted cristae in oocytes, and disrupted nuclei and disorganized cell membranes in granulosa in a dose-dependent manner. Data showed that cleavage and blastocyst rates in the 250 µg/ml of D-SPIONs were significantly lower than in the control group (P<0.05). Gene expression of *GPx1, Sod1, catalase, Bcl2l1* and *Bax* in resultant blastocysts of the high-dose group and catalase and Bax in resultant blastocysts of the low-dose group, was higher than the controls.

**Conclusion:**

There is considerable concern regarding D-SPIONs toxic effects on IVF, and mitochondrial and cell membrane damage in mouse spermatozoa and oocytes, which may be related to oxidative stress and apoptotic events.

## Introduction

Nowadays, there is great interest towards using nanotechnology due to its increasing application in all aspects of life, including agriculture, industry, medicine and public health (1, 2). All nanoparticles have a common characteristic: nanoparticle synthesis leads to remarkable changes in their chemical, physical and biological properties when compared to their original counterparts (3). Despite the beneficial properties of nanomaterials, potential risks of these materials are a matter of concern. Since some nanomaterials are used in medicine, there is concern about possible toxicity of these nanomaterials for human health (4, 5). Important toxicological concerns regarding the engineered nanomaterials are related to their redox potential, and transport of some particles across the biological cell membranes, particularly into the mitochondria (6). Toxicity of nanoparticles to the female reproductive system and fertility has been confirmed in some studies (7, 8). Likewise, titanium dioxide nanoparticle induced testis and sperm lesions, and diminished sperm numbers and sperm motility in male mice (9). Therefore, further studies are required to examine the biocompatibility and safety of these new materials in greater detail.

Superparamagnetic iron oxide nanoparticles (SPIONs) have magnetic, electronic and optical properties, which make them suitable for medical and scientific applications such as in vitro diagnostic tests, SPION-based contrast enhancement in magnetic resonance imaging, magnetic hyperthermia treatment and magnetic drug targeting for diagnosis and therapy of cancer and other diseases (10).

A considerable body of evidence indicates that SPIONs have toxic activities. Toxicity and reactive oxygen species (ROS) production in response to uptake of metal oxide nanoparticle, are caused by generation of hydroxyl radicals by strong catalytic impacts of nanoparticle surfaces such as content of iron oxide, and release of iron ions into an aqueous phase, and result in superoxide-driven Fenton reaction (11). Also, promotion of intracellular free iron levels leads to a ROS-antioxidant imbalance due to stimulation of ROS generation over Fenton and Haber- Weiss reactions. Consequently, SPION induces oxidative damage by a ROS -mediated mechanism (12) and leads to apoptosis by affecting the mitochondria, death receptors and endoplasmic reticulum. The mitochondrial pathway of apoptosis is mediated by the B-cell-lymphoma protein 2 (Bcl-2) family which includes two main groups: antiapoptotic (Bcl-2, Bcl2l1, Bcl-W, Bcl-B, A1 and Mcl- 1) and pro-apoptotic (Bax, Bak and Bok) proteins. Maintaining a balance between these groups is critical for cell protection against apoptosis (13).

Nanoparticle coating with biocompatible polymers such as chitosan or dextran, may act as a barrier against SPIONs’ toxic potential and hugely protect cellular molecules, such as lipids, proteins, and DNA, from oxidative stress (14). Such coating also increases the colloidal stability, aggregate size, cellular interaction and biocompatibility, and iron oxide cores (15). Thus, these polymers have dire effects on the fate and level of SPIONs uptake in different cells. Stroh et al. (16) showed that citrate-coated SPIONs could dramatically promote protein oxidation and oxidative stress, but do not affect cell viability.

Although our knowledge about SPIONs toxicity has improved in recent years, the effects of this nanoparticle on fertilization are still a major concern, because iron oxide nanoparticles have the capacity to penetrate the placenta and aggregate in the fetus (17). Moreover, small nanoparticles could cross the blood-testis barrier and appear in the testes (18). Thus, in this paper, the potential risks of D-SPIONs for murine in *vitro* fertilization (IVF) were investigated by transmission electron microscopy (TEM) in sperm, granulosa cells and oocytes. Then, the developmental competence and changes in antioxidant enzymes (glutathione peroxidase 1 (*GPx1*), superoxide dismutase 1 (*Sod1*) and catalase (*Cat*), *Bcl2l1* (apoptotic inhibitor) and Bax (apoptotic activator) gene expression, were evaluated in the resultant blastocysts.

## Materials and Methods

In this experimental study, all chemicals and reagents were purchased from Sigma Chemical Co. (St. Louis, USA) and Gibco (Grand Island, USA), unless stated otherwise.

### Preparation of dextran-coated nanoparticle suspension

The starting materials, FeCl_3_•6H_2_O, FeCl_2_•4H_2_O, and NH4OH solution were purchased from Merck. Magnetite nanoparticles were synthesized according to the literature with some modifications (19) through the alkaline coprecipitation method using iron (II) and (III) chlorides. Briefly, 1.6 g FeCl_3_•6H_2_O and 0.6 g FeCl_2_•4H_2_O were grinded and then added to a beaker containing 50 ml deionized water. The beaker was kept in an ultrasonic bath for 30 minutes. The prepared solution was transferred into a three-neck flask and agitated vigorously under nitrogen gas atmosphere. After 5 minutes’ agitation, 30 ml NH4OH was added dropwise during 45 minutes. Finally, the suspension was kept at 75-80°C for 80 minutes. The nanoparticles were separated magnetically and washed several times to adjust the pH. The collected iron oxide nanoparticles were dispersed in a 5% dextran solution and stirred for 5 hours at 75°C. The solution containing dextran-coated iron nanoparticles, was centrifuged at 11000 rpm for 15 minutes to eliminate the larger particles.

### Evaluation of D-SPIONs characterization

Phase analysis was performed by a Philips X-ray diffractometer (model PW3710) using Cu-Kα radiation at a wavelength of 1.54 A in the 2θ range of 5-80°. Fouriertransform infrared spectroscopy (FTIR) of the sample was done by a PerkinElmer spectrometer in the range of 400-4000 cm-1. TEM experiments were conducted on a Philips CM30 TEM with an operating voltage of 200 kV. The TEM sample preparation was done according to the literature (20). The nanoparticles containing aqueous solution, were sonicated and then, a drop of the solution was placed on the carbon-supported Cu grid. The nanoparticles on the grid, were used for the experiment after solvent evaporation.

### Animals

Fifty mature female BALB/c mice (6-8 weeks old) were superovulated by an intraperitoneal (IP) injection of 10 IU pregnant mare serum gonadotropin (PMSG, GONASER®, HIPRA, Amer, Spain) followed 48 hours later by injecting 10 IU human chorionic gonadotropin (hCG, Organon, Oss, The Netherlands). Sperm samples for IVF and TEM, were obtained from the caudae epididymides of fifteen mature (12-week-old) male BALB/c mice. All females and males were kept under controlled temperature and humidity conditions with a 12-hour light/dark schedule. Animals had *ad libitum* access to food and water. All animal care and procedures were approved by the Ethics Committee of Shiraz University of Medical Sciences (Approval No. IR.SUMS.REC.1396.S356).

### Experimental design

To evaluate possible toxic effects of D-SPIONs on sperm, granulosa cells, oocytes and resultant blastocysts, three groups were considered according to the level of D-SPIONs added to IVF medium [G-IVF PLUS (Vitrolife, Gothenburg, Sweden)]; Group I (control), conventional IVF medium without any treatment (G-IVF PLUS); Group II and Group III conventional IVF medium supplemented with 50 μg/ml and 250 μg/ml of D-SPIONs, respectively. In all groups, sperm or COCs were incubated for 4 hours in IVF medium and fixed with glutaraldehyde for electron microscopic analysis. To evaluate the effects of this nanoparticle on developmental competence, IVF was done in G-IVF PLUS supplemented with 0, 50 and 250 μg/ml of nanoparticles. Then, the presumptive zygotes were cultured until expanded, reaching the blastocysts stage in G1/G2 PLUS without nanoparticle. The resultant expanded blastocysts were used for gene expression analysis.

### Sperm capacitation

Spermatozoa were collected from the cauda epididymides of mature male mice and capacitated by preincubation at 37°C with 5% CO_2_ for 1 hour in 200 μl of G-IVF PLUS drops under mineral oil (Reproline Medical GmbH, Rheinbach, Germany). These spermatozoa were used for IVF and TEM assay.

### Sperm preparation for TEM

Spermatozoa were randomly divided into three groups and incubated for 4 hours in G-IVF medium with different concentrations (0, 50 and 250 μg/ml) of nanoparticles under mineral oil. After incubation, 0.5 ml of semen was transferred into a micro tube, washed with phosphate buffered saline (PBS) twice and centrifuged at 400 g for 10 minutes. Then, the supernatant was removed and each sample was fixed with 2.5% glutaraldehyde (pH 7.4) overnight. The samples were centrifuged for 10 min at 400 g at room temperature and washed in sodium cacodylate for 3 times (5 minutes each) and centrifuged for 10 minutes at 400 g. Each sample was post-fixed in 1% buffered osmium tetroxide for 60 minutes. Post-fixed samples were centrifuged for 10 minutes at 400 g, and the supernatant was discarded; then, samples were washed in sodium cacodylate for 3 times (5 minutes each) and embedded in 1% agar. After that, embedded samples were dehydrated in ascending concentrations of 30-100% ethanol. Finally, the samples were embedded in resin (agar 100) and polymerized at 60˚C overnight. Thick sections (0.5-1 μm) were stained with toluidine blue and examined by light microscope (Axioskop, Carl Zeiss Microscopy GmbH, Gottingen, Germany). Thin sections (60-90 nm) were contrasted with uranyl acetate and lead citrate and examined by TEM (21).

### COCs collection

The COCs were immediately harvested from the oviductal ampulla 13-14 hours post-hCG injection. These COCs were used for IVF and TEM assay.

### COCs preparation for TEM

COCs were exposed to different concentrations (0, 50 and 250 μg/ml) of nanoparticle in the G-IVF medium under mineral oil for 4 hours at 37°C with 5% CO_2_. Then, they were washed twice in PBS to remove culture medium and nanoparticles. COCs were immersed in 2.5% glutaraldehyde overnight. Then, COCs were washed in sodium cacodylate for 3 times (5 minutes each). Following fixation in 1% buffered osmium tetroxide for 30 minutes, COCs were washed in sodium cacodylate for 3 times (5 minutes each) and dehydrated in ascending concentrations of 30-100% ethanol. Each COC was embedded in resin (agar 100) and polymerized at 60°C overnight. Thick sections (0.5-1 ىm) were stained with toluidine blue and examined by light microscope. Thin sections (60-90 nm) were contrasted with uranyl acetate and lead citrate and examined by TEM (21).

### *In vitro* fertilization and embryo culture

COCs were inseminated in vitro with 1×10^6^ spermatozoa/ml in 100 μl of G-IVF PLUS containing 0, 50 or 250 μg/ml of D-SPIONs, for 4 hours. The presumptive zygotes were cultured in G1 PLUS for 1.5 days, and then, the embryos were transferred to G2 PLUS under mineral oil at 37˚C in a humidifi ed incubator with 5.0 % CO_2_, and the rates of cleavage and blastocyst were recorded in at least 4 replicates.

### RNA extraction, cDNA synthesis and quantitative real-time RT-PCR

Total RNA was extracted from 3 pools of 15 expanded blastocysts per group, using the RNeasy Micro Kit (Qiagen, Hilden, Germany) following the manufacturer instructions. First-strand cDNA synthesis was carried out using the QuantiTect Reverse Transcription Kit (Qiagen, Hilden, Germany) according to the manufacturer’s instructions. Real-time reverse transcription - polymerase chain reaction (RT-PCR) was performed using an ABI Prism 7500 Sequence Detection System (Applied Biosystems, Foster City, USA). The PCR amplification was conducted in a final volume of 25 μl consisting of 1 μl of the cDNA template, 12.5 μl of RealQ Plus 2x Master Mix Green Low ROX (Ampliqon A/S, Odense, Denmark), and 1 μl of each primer (10 pmol/μl). Glyceraldehyde-3-phosphate dehydrogenase (*Gapdh*) was used as a reference (22). The gene expression of *GPx1*, *Sod1* and *Cat* as main antioxidant enzymes, and *Bcl2l1* and *Bax* in expanded blastocysts, was analyzed using the 2^-∆∆Ct^ method. The primers used for RT-PCR are listed in Table 1.

**Table 1:** Details of primers used for quantitative real-time reverse transcription - polymerase chain reaction (RT-PCR)


Gene	Nucleotide sequences (5′–3′)	Fragment size (bp)	Accession number

*GPx1*	F: CAGGAGAATGGCAAGAATGAAGAG	136	NM_008160.6
	R: GGAAGGTAAAGAGCGGGTGA		
*Sod1*	F: GGGTTCCACGTCCATCAGTAT	121	NM_011434.1
	R: GGTCTCCAACATGCCTCTCTT		
*Cat*	F: CTCAGGTGCGGACATTCTACA	206	NM_009804.2
	R: AATTGCGTTCTTAGGCTTCTCAG		
*Bcl2l1*	F: GCAGGTATTGGTGAGTCGGA	130	NM_001289716.1
	R: CTCGGCTGCTGCATTGTTC		
*Bax*	F: TGGAGATGAACTGGACAGCAAT	155	NM_007527.3
	R: TAGCAAAGTAGAAGAGGGCAACC		
*Gapdh*	F: TGTTTCCTCGTCCCGTAGA	106	NM_001289726.1
	R: ATCTCCACTTTGCCACTGC		


### Statistical analysis

Before any statistical analysis, the normality of data and homogeneity of variances were evaluated by the Shapiro- Wilk test and means of Bartlett’s test, respectively. Developmental competence and real-time RT-PCR data were analyzed by one-way ANOVA followed by Tukey’s multiple comparison test using SPSS 20 (IBM Corp., Armonk, N.Y., USA). Data is expressed as mean ± standard deviation (SD). Differences were considered significant at P<0.05.

## Results

### D-SPIONs characterization

D-SPIONs were characterized by X-ray Diffraction (XRD) pattern, TEM and FTIR spectrum (Fig . 1). The XRD pattern of the synthesized nanoparticles showed that all the peaks corresponded to Fe3O4 and no other peak from other phases, could be detected. In XRD analysis, the major XRD peak was calculated at 2θ = 35.6 and other peaks were observed from 0 0 1, 1 1 2, 1 0 3, 0 0 4, 2 0 4, 3 2 1, 2 2 4 and 4 1 3. The full width at half maximum (FWHM) of the 1 0 3 peak was used to estimate the average crystallite size of D-SPIONs using the Scherrer method. The average size of D-SPIONs was 17.44 nm.

**Fig.1:**
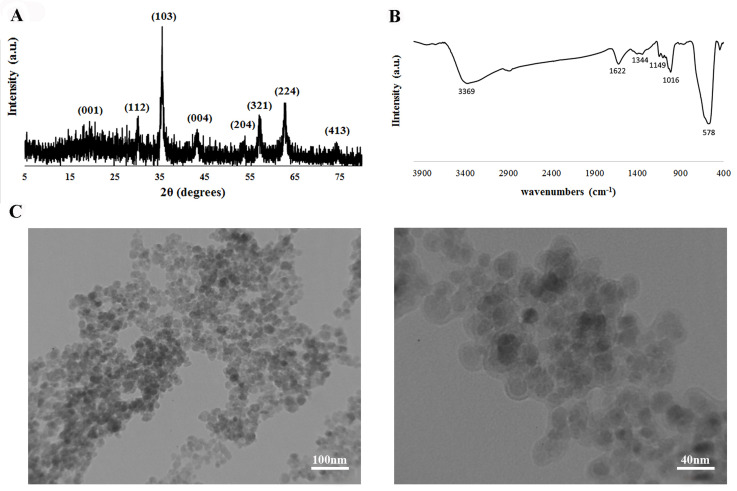
Evaluation of dextran-coated superparamagnetic iron oxide nanoparticles (D-SPIONs) characterization. **A.** XRD pattern of the synthesized D-SPIONs shows that all peaks correspond to the magnetite. **B.** FTIR spectrum for synthesized D-SPIONs. **C.** TEM images of D-SPIONs show that the sample consists of core/shell monodispersed nanoparticles with particle size in the range of 20-30 nm.

The FTIR spectrum of the synthesized D-SPIONs, is shown in Figure 1B. The peak at 578 cm^-1^ corresponded to the Fe-O bond absorption. The peak at 1622 cm^-1^ is an indication of C=O stretching vibrations. Peaks at 1016 cm-1 and 1149 cm^-1^ corresponded to the C-OH alcoholic hydroxyl stretching vibrations and the peak at 3380 cm^-1^ showed the presence of the hydroxyls in the dextrancoated nanoparticles (23). The bands seen around 2900 cm^-1^ and 1240-1460 cm^-1^, showed the νC–H and the δC–H vibrational modes of the dextran (24).

The TEM images of D-SPIONs are presented in Figure 1C. The sample consisted of monodisperse coated nanoparticles with particle size in the range of 20-30 nm. The coating of the particles was almost uniform with rounded shapes which might result in better biocompatibility.

### D-SPIONs destroyed the mitochondria and membranes of spermatozoa in a dose-dependent manner

**Fig.2:**
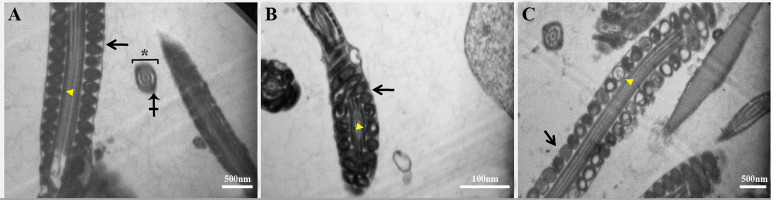
Transmission electron microscopy (TEM) analysis in sperm. **A.** Control group with intact cell membranes (arrow) and mitochondria with normal shape (arrowhead). Axoneme (asterisk) and longitudinal fiber microtubules (crossed arrow) in tail regions are normal. **B.** In 50 μg/ml D-SPIONs-treated group, some of the sperm mitochondria are swollen (arrowhead) but cell membrane was normal (arrow). **C.** Due to internalization or binding of D-SPIONs in the high dose group (250 μg/ml), most mitochondria are swollen (arrowhead), have entirely distorted mitochondrial cristae, and the cell membrane in the midpiece is disorganized and/or distorted (arrow).

The degree of D-SPIONs effect was clearly dependent on their concentration. After co-incubation with D-SPIONs, the nano-treated spermatozoa and control sperm cells were subjected to TEM. As shown in Figure 2, some of the sperm mitochondria in the low-dose (50 μg/ml) D-SPIONs group, were swollen but cell membrane was normal. Most of the spermatozoa mitochondria in the high -dose (250 μg/ml) D-SPIONs group, were swollen, with completely distorted mitochondrial cristae, and cell membrane in the midpiece was disorganized and/or distorted, whereas in the control group, spermatozoa mitochondria were regular in shape. Axoneme and longitudinal fiber microtubules in the tail regions were normal.

### D-SPIONs had negative effects on oocyte mitochondria and nuclei and membranes of granulosa cells in a dosedependent manner

**Fig.3:**
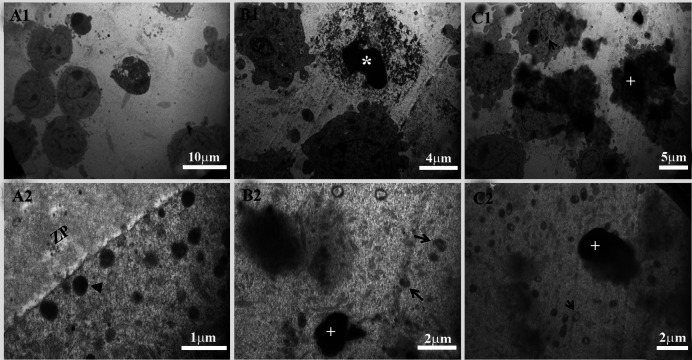
TEM analysis in granulosa and oocyte. **A1.** Normal granulosa cells in the control group. **B1.** Some granulosa cells in 50 μg/ml dextran-coated superparamagnetic iron oxide nanoparticles (D-SPIONs), with dense nuclei and disorganized and/or distorted cell membranes (star). **C1.** Most of the granulosa cells in 250 μg/ml D-SPIONs with disrupted nuclei show disorganized cell membranes (arrow) and aggregation of nanoparticles between the granulose cells (plus). **A2.** Mitochondria with normal shape and cortical granules (arrowhead) in the oocytes of the control group. **B2.** Ooplasm in 50 μg/ ml D-SPIONs has few vacuolated and cristae-distorted mitochondria and several normal mitochondria (arrow). **C2.** Ooplasm in 250 μg/ml D-SPIONs have vacuolated and cristae-distorted mitochondria (arrow). The plus sign denotes D-SPIONs. ZP; Zona pellucida.

As the nanoparticles’ dose increased, the effect of nanoparticles on the granulosa cells and mitochondria in the ooplasm, became more obvious. As shown in Figure 3, some granulosa cells in the low-dose (50 μg/ml) D-SPIONs group, had dense nuclei and cell membranes were disorganized and/or distorted. Most of the granulosa cells in high-dose (250 μg/ml) D-SPIONs group, had disrupted nuclei, disorganized cell membranes and aggregation of nanoparticles, clearly discerned between the granulose cells. In the ooplasm, mitochondria were vacuolated and cristae were distorted, whereas in the control group, mitochondria had regular shape and cortical granules were seen.

### D-SPIONs reduced oocyte developmental potential in the high-dose group

The developmental competence of MII oocytes after D-SPIONs treatment, was evaluated by IVF and culture in G1/G2 media until the blastocyst stage. As shown in Table 2, the rate of embryo cleavage at 250 μg/ml of D-SPIONs was significantly lower than that of the control group, with 89.79 ± 2.68%, 76.62 ± 6.10%, and 69.79 ± 6.15% (P<0.05) in the control, and 50 and 250 μg/ml of D-SPIONs, respectively. The proportion of oocytes that developed to the blastocyst stage, was significantly lower (P<0.05) at 250 μg/ml of D-SPIONs (34.99 ± 11.42%) compared to the control group (67.08 ± 4.90%).

**Table 2:** Effect of different concentrations of D-SPIONs in IVF medium, on developmental potential


D-SPIONs(μg/ml)	No. of oocytes	Cleavage raten (% ± SD)^1^	Blastocyst formationn (% ± SD)^1^

0	157	139 (89.79 ± 6.00)	101 (67.08 ± 10.95)
50	269	214 (76.62 ± 12.21)	146 (51.92 ± 13.63)
250	240	157 (69.79 ± 12.30)^*^	81 (34.99 ± 22.83)^*^


^1^; The ratio of cleavage and blastocyst embryos per MII oocytes from at least 4 replicates, ^*^; Mean percentage marked by an asterisk in each column, is significantly different from the control group (P < 0.05), SD; Standard division, and IVF; *In vitro* fertilization.

### High-dose D-SPIONs increased the expression of antioxidant enzyme genes

**Fig.4:**
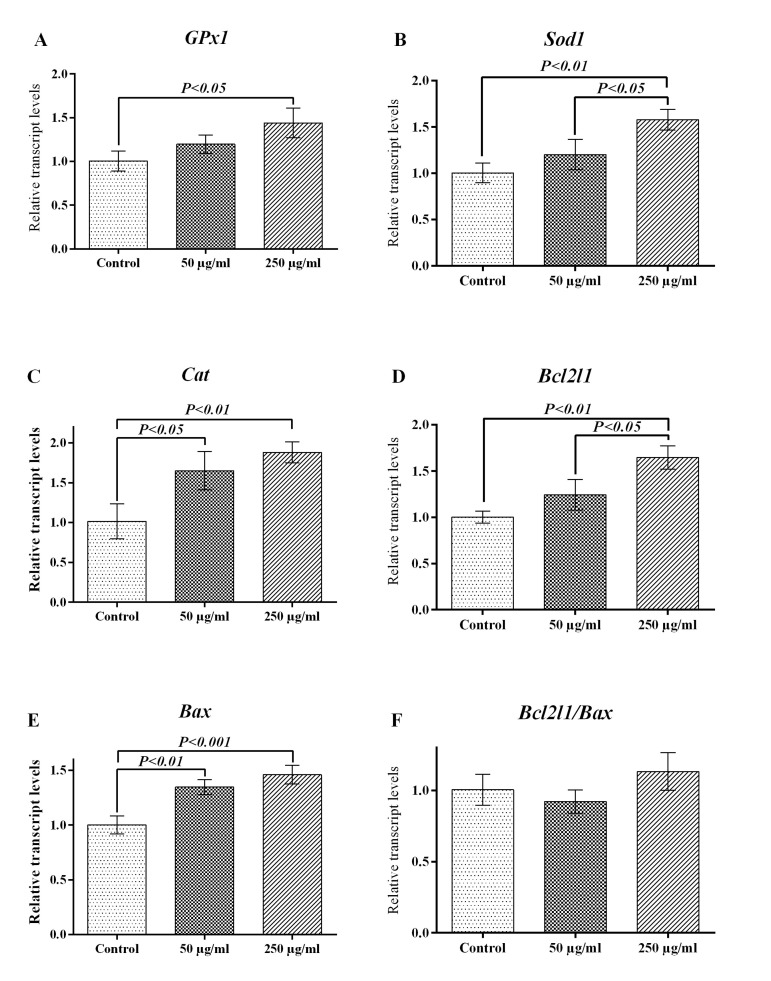
Addition of dextran-coated superparamagnetic iron oxide nanoparticles (D-SPIONs) to the in *vitro* fertilization medium alters gene expression of antioxidant enzymes and apoptotic genes in resultant expanded blastocysts. **A.** Glutathione peroxidase 1 (*GPx1*), **B.** Superoxide dismutase 1 (*Sod1*), and **C.** Catalase (*Cat*) are the main antioxidant enzymes. **D.** *Bcl2l1* and **E.** *Bax* are apoptosis inhibitor and activator, respectively, and **F.** Is ratio of *Bcl2l1* to *Bax*.

In order to investigate whether the addition of D-SPIONs to the IVF medium changes the expression of three main antioxidant enzymes, we quantified the transcripts of glutathione peroxidase 1 (GPx1), superoxide dismutase 1 (Sod1) and catalase (Cat) genes in each group on the expanded blastocysts. As shown in Figure 4, transcript levels of the GPx1 gene were significantly increased by 250 μg/ml of D-SPIONs (1.44 ± 0.10) when compared to the control group (1.00 ± 0.07, P<0.05). The result of real-time RT-PCR indicated that the relative Sod1 mRNA expression was upregulated by 250 μg/ml of D-SPIONs (1.58 ± 0.06) in the expanded blastocysts, compared with 50 μg/ml of D-SPIONs (1.20 ± 0.09, P<0.05) and the control group (1.00 ± 0.06, P<0.01). Transcript abundance of Cat was significantly increased in 50 and 250 μg/ml of D-SPIONs groups (1.65 ± 0.14, P<0.05 and 1.88 ± 0.08, P<0.01, respectively) in comparison to the control group (1.02 ± 0.12).

High levels of Bcl2l1 transcript were observed in 250 μg/ml of D-SPIONs group (1.65 ± 0.07) when compared to 50 μg/ml of D-SPIONs (1.24 ± 0.10, P<0.05) and control (1.00 ± 0.04, P<0.01) groups. Gene expression of Bax as an apoptotic activator was promoted in both of the D-SPIONs groups (1.35 ± 0.04, P< 0.01 and 1.46 ± 0.05, P< 0.001 for 50 and 250 μg/

 ml of D-SPIONs group, respectively) compared to the control group (1.00 ± 0.05), the ratio of Bcl2l1 to Bax was not significantly different among the groups (P>0.05).

## Discussion

Despite the potential benefits of nanoparticles, some literature has demonstrated that nanoparticles might have negative impacts on biological systems based on their size and properties (25). Although several surface modifications have been applied to make these nanoparticles more biocompatible, their potential toxic effects remain a matter of concern. The purpose of this investigation was to evaluate the interaction of D-SPIONs and sperm, oocyte and granulosa cells, as well as the probability of changes in oxidative stress enzymes and apoptotic genes in resultant blastocysts in a dose-dependent manner (50 and 250 μg/ml) in an *in vitro* mouse model.

TEM in the midpiece sperm, which is responsible for the vigor of sperm, revealed that D-SPIONs destroyed most of the spermatozoa mitochondria and membranes at high dose (250 μg/ml), while a few of the sperm mitochondria and no membranes were affected by low dose (50 μg/ ml) D-SPIONs. Jeng and Swanson (26) also revealed that high concentration of SPIONs had a negative e-ect on mitochondrial function. Oral administration of high dose (200 mg/kg/day) polyvinyl pyrrolidone-coated silver nanoparticles could induce adverse effects on sperm morphology (18). It has been indicated that swelling of the midpiece and mitochondrial enlargement led to a disruption in redox metabolism, enhancement of ROS generation and induction of apoptosis (27). Similar to our findings for sperm, our results indicated that disruption in oocyte and granulosa cell was directly correlated with dose-dependent increases in D-SPIONs. Liu et al. (28) reported that calcium phosphate nanoparticles could penetrate human granulosa cells, and enter lysosome and mitochondria. Likewise, Courbiere et al. (29) in their study on the mouse oocyte, showed that cerium dioxide nanoparticles were capable of penetrating the oocyte zona pellucida, and the accumulation of nanoparticles led to in vitro toxicity. Another study found toxic effects of cerium dioxide nanoparticles on mouse spermatozoa and oocytes (30). These studies and our results contrast a previous investigation which reported that human granulosa cells (HLG-5) treated with different coatings of SPIONs showed no toxic effects and results indicated ameliorated biocompatibility properties (31). This discrepancy may be related to the kind and concentration of nanoparticles, species and experimental condition.

The results of the present study showed that exposure of sperm and oocyte to D-SPIONs for 4 hours in fertilization medium, caused a significant decrease in cleavage and blastocyst rates in a concentration-dependent manner. This ﬁnding was in agreement with a previous study showing a significant reduction in cleavage rate by treatment of fertilization culture with cerium dioxide nanoparticles (30). Hsieh et al. (32) reported adverse impacts of CdSecore quantum dots (QDs) on mouse oocyte maturation, and fertilization and on embryo early development, but that was not the case for ZnS-coated CdSe QDs. They concluded that surface modification of CdSe-core QDs with ZnS, significantly inhibits their toxicity. In contrast, our results showed that surface modification of D-SPIONs with dextran could not effectively prevent the negative impacts of this nanoparticle. It seems that the oxidative stress response to SPIONs could be produced by at least four sources: 1. generation of ROS from the surface of this nanoparticle, 2. production of ROS via leaching iron ions from the surface degradation, 3. disrupting mitochondrial and other organelle functions, and 4. induction of cell signaling pathways which triggered the production of ROS (33). Thus, as explained above, these mechanisms, by generation of ROS, could influence fertilization. Oxidative stress not only promoted lipid peroxidation by damaging the cell membrane (34), but also induced DNA fragmentation in sperm which triggered a reduction in fertilization rate. Sperm DNA damage led to a disruption in zona pellucida binding which subsequently resulted in a low rate of fertilization (35). Furthermore, oxidative stress induced by nanoparticles is associated with DNA damage and had a negative effect on oocyte quality in mouse oocyte (29).

In our study, the levels of *GPx1*, *Sod1* and catalase transcripts as antioxidant enzymes, in the high dose group were significantly higher than that of the control group in resultant blastocysts. It has been demonstrated that *GPx1* is related to lipid peroxidation. *GPx1* and *Sod1* have an important role in the spermatozoa membrane integrity (36). Interaction between iron and some free radicals such as superoxide through the Haber - Weiss reaction leads to production of highly toxic hydroxyl radicals (12). Thus, these results may suggest that after exposing the oocyte and sperm to SPIONs, these antioxidant enzyme genes, as a ROS scavenger, significantly increased over time to protect the resultant embryos from oxidative stress. Another possible reason may be related to higher mitochondria dysfunction in the high-dose D-SPIONs group. It has been demonstrated that upregulated *GPx1* also leads to mitochondrial dysfunction, and a reduction in cellular proliferation, mitochondrial potential and ATP production. Thus, GPx1, by regulating mitochondrial function, may moderate redox-dependent cellular responses (37).

This study, surprisingly, showed a significant increase in the anti-apoptotic *Bcl2l1* gene in the high-dose D-SPIONs group when compared to the low-dose and control groups, while pro-apoptotic *Bax* gene expression in both nanoparticle groups was significantly higher than that of the control group. The *Bcl2l1*/*Bax* ratio in this study was not significantly different among groups. BCL- 2 family proteins play an important role in regulating the mitochondrial-related apoptosis pathways; it seems that surviving blastocysts with promotion of mitochondrial antioxidant enzymes, upregulation of *Bcl2l1* and maintenance of *Bcl2l1*/*Bax* ratio, prevented DNA damage and cell death. Ilani et al. (4) by IP administration of titanium dioxide nanoparticles in female mice, found that rates of fertilization and blastocysts were not affected; however, levels of *Bcl2l1* and Bax expression respectively decreased and increased by titanium dioxide nanoparticles, which may be related to the apoptotic effect of this nanoparticle in resultant blastocysts. It has been confirmed that *Bcl2l1* prevents apoptosis by binding to the BH3 domains of BAX and BAK1 to prevent their activation (38), therefore, probably in response to *Bax* overexpression, the amount of *Bcl2l1* increased to inhibit apoptotic effects of Bax.

## Conclusion

This study, for the first time, found that despite massive use of D-SPIONs in various fields of science such as medicine, considerable concern exists regarding their toxicity towards IVF, and mitochondrial and cell membrane damage in mouse spermatozoa and oocytes, as well as overexpression in oxidative enzymes and apoptotic genes in the resultant blastocysts. Therefore, it is beneficial to examine possible toxicity of this nanoparticle before its application in various fields of nanotechnology. Future studies are needed to understand more details about the mechanisms and molecular pathways of interaction between D-SPIONs and reproductive cell damage. It is also essential to evaluate its biocompatibility and possible toxic effects on other cells, tissues and organs.
